# Sustainability Certification, a New Path of Value Creation in the Olive Oil Sector: The ITALIAN Case Study

**DOI:** 10.3390/foods10030501

**Published:** 2021-02-26

**Authors:** Luca Lombardo, Camilla Farolfi, Ettore Capri

**Affiliations:** 1 Università Cattolica del Sacro Cuore, Department for Sustainable Food Process, Via Emilia Parmense 84, 29122 Piacenza, Italy; 2 European Observatory on Sustainable Agriculture (OPERA), Università Cattolica del Sacro Cuore, Via Emilia Parmense 84, 29122 Piacenza, Italy; camilla.farolfi@unicatt.it (C.F.); ettore.capri@unicatt.it (E.C.)

**Keywords:** sustainability, olive oil, Italian oliviculture, common agricultural policies, certifications

## Abstract

The Italian extra virgin olive oil supply chain has considerable potential for embarking on a path of sustainable development and evolution. In Italy, the great variety heritage and the different pedo-climatic characteristics result in local olive growing systems with different management techniques, producing extra virgin olive oils that are strictly entwined to the territory, with peculiar qualitative properties. Nevertheless, numerous criticalities have been traditionally eroding the competitiveness of Italian olive growing that could find in sustainability certifications, a lasting driver of value creation. Shared standardizations and certifications that include the three pillars of sustainability are therefore necessary for the development of the process.

## 1. Introduction

The need to address at the intergovernmental level the issue of how to achieve a stable economic growth that was at the same time environmentally sustainable dates back to the late 1960s, with the UNESCO’s “Biosphere Conference” and the “Conference on the Ecological Aspects of International Development”, both held in 1968 [1]. These concepts were further developed during the 1972 United Nations Conference on the Environment in Stockholm, while in the “World Conservation Strategy” report by the International Union for the Conservation of Nature [2] there was the first written reference to sustainable development in its modern acceptation. In 1981, Spreckley [3] argued that enterprises should incorporate in their performance assessment: “financial performance, social wealth creation and environmental responsibility”. In 1987, Barbier [4] first schematized sustainable economic development (compared with conventional and Marxist economics) as the intersection of the biological and resource system, the economic system and the social one. In the same year, the Brundtland Report [5] provided the well-known definition of sustainable development as: “development that meets the needs of the present without compromising the ability of future generations to meet their own needs”. During the United Nations Conference on Environment and Development (UNCED) held in Rio de Janeiro, from 3 to 14 June 1992, 178 Governments voted to adopt the Agenda 21 program, the non-legally binding declaration on sustainable development, and the principles of sustainable forest management. Eventually, the 2030 Agenda for Sustainable Development, signed in September 2015 by 193 UN member countries, set 17 objectives (Sustainable Development Goals—SDGs) divided into 169 targets aimed at ending poverty, safeguarding the planet, ensuring welfare [6].

Nevertheless, the difficulty in jointly defining global guidelines (often due to the failures of multilateral negotiations), the ineffectiveness or absence of regulations by individual governments, and the rise in the demand for sustainability-certified products, were the driving force at birth of non-state market-driven (NSMD) governance systems. These involved private companies and non-governmental organizations (NGOs) [7,8,9]. Their purpose was to develop and implement environmental and social equitability standards (Voluntary Sustainability Standards—VSS), with a clear reference to the three pillars of sustainable development. VSS had to be respected in the production process and required compulsory verification of compliance through a third-party certification, guaranteed by a labeling system. In this sense, the certification program Oregon Tilth Certified Organic (OTCO), established in 1982, was the first attempt at a market incentive through labeling of organic food [10]. Since then, according to the Ecolabel Index [11], there are 456 environmental and social voluntary sustainability standards and labels (the most common of which are reported in Table 1) in 199 countries, and 25 industry sectors and are increasingly recognized as potentially transformative tools for increasing consumer awareness, expanding the market for niche products and for urging governments to realize their sustainability commitments [12]. As a consequence, standardization and conformity assessment of voluntary environmental declarations are focal points for the creation of a framework for sustainability labeling, which includes the nutritional, climatic, environmental, social, and economic aspects of food products. This might be particularly true in the case of olive oil, where the nutritional value, the link with the territory, the environmental and social responsibility are an added value and a marketing tool to both attract and protect consumers. To implement a sustainability certification process, it is necessary to verify and establish the areas of intervention in the extra virgin olive oil production chain. Furthermore, it is necessary to know the economic and social substratum of the nation’s olive growing system. Accordingly, the Italian olive oil sector is characterized by numerous criticalities and problems, which must necessarily be solved in order to develop a sustainable and profitable supply chain. In the present paper, we present an analysis of: i) the environmental hotspots in the sustainability certification process for the olive oil sector; ii) strengths and weaknesses of the Italian oliviculture and how a certified system of sustainability could induce a lasting economic and social development; iii) the sustainability certifications actually implemented in Italy in the olive oil sector.

## 2. Environmental Sustainability Assessment of Olive Growing Systems and Standardization of Certification

In order to allow a homogeneous comparison of the environmental impacts of the same product or service, it is necessary to establish shared product category rules (PCR) the different producers must comply with when conducting the life cycle analysis (LCA). The European Commission’s Environmental Footprinting (EF) pilot phase drew up the Product Environmental Footprint Category Rules (PEFCR) for olive oil [13], compliant with the Product Environmental Footprint (PEF) Guide; Annex II to the Recommendation 3312013/179/EU, 9 April 2013. The objective was to provide clear guidelines for developing PEFCRs and OEFSRs through life cycle assessment according to the ISO 14040 and 14044 standards [14,15]. Nevertheless, olive oil pilot was postponed to the transition phase (namely the period between the end of the Environmental Footprint pilot phase and the possible adoption of policies implementing the PEFCR).

In the private sector, a Product Category Rules (PCR) for Environmental Product Declaration (EPD) document was created for the Italian olive oil producer Apolio for the extra virgin olive oil “Denocciolato” [16]. An Environmental Product Declaration (EPD) according to the International EPD^®^System, is an independently verified and registered document that communicates standardized information on the environmental impact of products through a life-cycle assessment in accordance with the international standard ISO 14025 (Type III Environmental Declarations). The PCR considered 3 phases: (1) Upstream processes (from cradle-to-gate or farm gate; the agricultural phase); (2) Core processes (from gate-to-gate or farm gate to mill gate, the processing phase); (3) Downstream processes (from gate-to-grave; the end-of life phase).

Several scientific studies related to the environmental performance of the olive oil sector based on the LCA methodology have been performed following a from “cradle to grave” approach, or, more often, a “cradle to farm gate” study. This because the agricultural phase is generally identified in the scientific literature as the most impactful, particularly because of fertilization, pesticides, and water management, whereas waste management represents a further crucial hotspot. The processing phase seems to be the less variable stage when comparing the different studies [17].

In a 2019 study [18], climate change impacts represented about 4% of total impacts as a result of the electricity consumption during the extraction phase. On the other side, positive impacts are generated through wastewater treatment. According to Rinaldi et al. [19], environmental criticalities must be identified in the distribution phase whenever air transport is chosen. Secondary hotspots are represented by fertilization, olive oil storage, and glass bottles manufacturing process.

In a comparative LCA-based study on the European olive production systems [20], Italy, showed the highest use of fertilizers leading to a higher total global warming, while in Spain, the highest use of organo-phosphorous pesticides led to the highest impacts of eco-toxicity. 

Finally, regarding olive grove management, organic farming commonly results to be less impactful than conventional and integrated ones [21,22,23,24].

As an analogy to the environmental footprint concept, two specific indicators have been introduced in response to the raising concerns regarding freshwater use and greenhouse gas emissions and global warming: water footprint and carbon footprint [25]. Their evaluation is based on the LCA methodology, and although they have been calculated through different methods, they are now regulated by international standards.

### 2.1. Water Footprint

Water footprint (WF, reference standard ISO 14046) is a multidimensional indicator of the total volume of fresh water directly and indirectly used by a consumer or a producer to produce goods and services. Water use is measured in water volume consumed (evaporated or incorporated into a product) or polluted per unit of time. WF is globally calculated as the sum of three components [26]:

− Blue water: the global surface and underground water intended for agricultural, domestic, and industrial use;
− Green water: the volume of rainwater that does not contribute to surface runoff and mainly refers to the water used by crops to grow;
− Grey water: the volume of polluted water generated during a production process, it represents the volume of fresh water needed to dilute the pollutants till the natural concentrations of the water quality standards [27].

With regard to the studies in the olive sector, according to Dichio et al. [28] green WF accounted for about for 48 and 90% in Italian irrigated and rain-fed systems, respectively, while for Salmoral et al. [29] in Spanish olive oil production green WF ranged from 72% in rain-fed systems to 12% in irrigated olive orchards, with blue and grey components representing 6 and 10% of the total WF. Similarly, in the comparison of WF of different olive agronomic cropping systems in Apulia Region [30] WF_green_ accounted for 65% and WF_blue_ for 24%, in the rainfed Traditional System (TS), whereas in the Intensive System (IS) and High-Density System (HDS), WF_blue_ resulted (for both irrigation and fertilizer production) to be the predominant fraction, about 77 and 74%, respectively, with WF_grey_ representing around 3%. Wide variability in these values was described by Amicarelli et al. [31] depending on cultivation techniques, different soil and climate condition: 6–40% for WF_green_; 15–35% for WF_blue_ (attributable to the different irrigation and fertilization practices); and 45–55% for WF_grey_, (mainly due to fertilizers production and application).

### 2.2. Carbon Footprint

Carbon footprint (CF, reference standard ISO 14067) is the total amount of greenhouse gas (GHG) expressed as carbon dioxide equivalent (CO_2eq_), directly or indirectly associated with a product, an individual, an organization, or a service.

**Figure 1:**
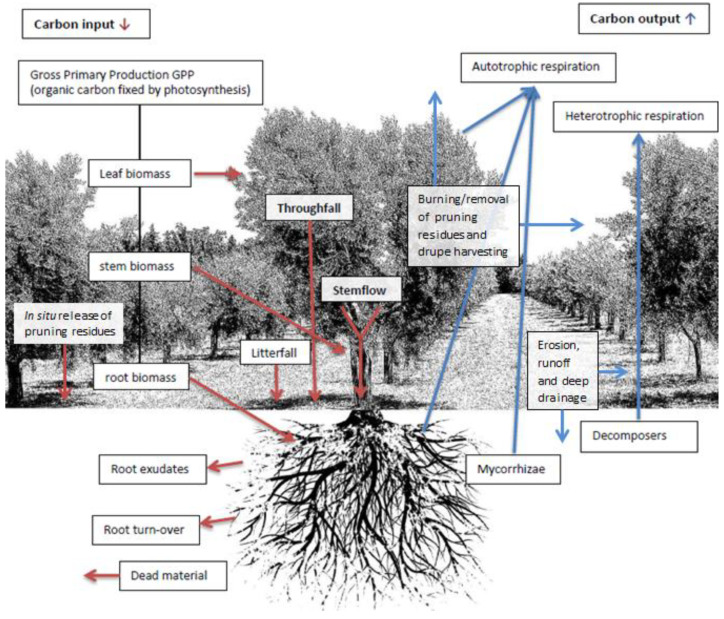
Carbon inputs and outputs in a typical olive grove in production.

Olive groves have been proved to be efficient atmospheric carbon sinks, as carbon inputs are generally higher than C outpust (Figure 1) thus playing a positive role in climate change mitigation [32,33]. In a two-year trial [34], 93% of the total carbon uptake (12.5 Mg C ha^−1^ year^−1^) measured within an intensive olive grove, accumulated in plant organs, with photosynthesis and respiration representing about 99% of the whole C cycle. In accordance, Proietti et al. [35] described olive trees capacity to store 28.916 kg CO_2_ year^−1^ plant^−1^. Considering more specific partitions of carbon input and output (Figure 1), according to Lombardo et al. [36] carbon fluxes via throughfall and stemflow in a conventional Spanish olive orchard were comparable to the average organic carbon losses due to sediment and run-off (9.2 g C/m^2^/year) [37], and water erosion (2.58 ± 0.66 g C/m^2^/year) [38] in different olive groves. Furthermore, the recycling of olive oil extraction by-products like virgin or exhausted olive pomace as biofuels can represent an effective strategy for GHG reductions [39].

Regarding the global warming potential over 100 years (GWP100, the contribution of a greenhouse gas to the greenhouse effect compared with the effect of CO_2_ over that period), the highest value was found [40] in the first year of cultivation. The breakeven point between sequestration and emission was measured after the fourth year, with the value of sequestration becoming 5–6 times greater than emissions after the tenth year.

## 3. Economic and Socio-Cultural Sustainability of Italian Olive Growing

Italy is currently the world’s second biggest olive producer (2,086,418 Mg) [41] and the first olive oil consumer (~509,000 Mg, average of the 2015 to 2019 period) [42]. As olive oil production (325,285 Mg, average of the 2015 to 2019 period) [43] fails to cover domestic consumption, Italy is also the first olive oil importer (~515,000 Mg) [44] and, as a result of these large imports, the second major exporter (~330,000 Mg), for a volume of business of ~1.2 billion euros (average of the 2015 to 2019 period) [43,45]. From an economic point of view, the olive sector accounts for 2.4% of national agri-food industry turnover [43]. Nevertheless, the Italian olive sector daily faces structural problems that dramatically limit its sustainable economic development, leading to loss of competitiveness and market share to its principal competitors (Spain, Tunisia, Greece, and Portugal). First of all, Italian oliviculture suffers of excessive fragmentation witnessed by approximately 825,000 farms operating in the sector with an average surface of 1.4 ha, served by 4480 authorized mills [43], and by the presence of 108 producer organizations (PO) −13 for wine sector- and 3 associations of producer organizations (APO) [46]. A further anomaly in the productive fabric is given by the fact that while 82% of olive production takes place in the Southern Regions, over 50% of the bottling plants are located in the Center–North of Italy, where, therefore, most of the imported oil arrives. Paradigmatic is the case of Tuscany that against a 5% production of Italian olive oil, it holds 45% of the value of outgoing foreign trade [43,45]. Additionally, only 22% of olive groves are found in plains (67% in hilly areas and 11% in mountains) [47] and only 14% of the orchards are irrigated [48], factors limiting the diffusion of super-intensive farming. 

A recent problem was the outbreak of *Xylella Fastidiosa* subsp. pauca strain CoDiRo in Apulia since 2013, causing a dramatic drop of olive production from ~1,150,000 Mg in the 2006 to 2013 period to ~805,500 Mg in the 2014 to 2020 years in the region [41].

Eventually, Legislative *Decree* n. 475/1945 (ratified by the Decree of the President of the Republic n. 987/1955), establishes that the felling of more than five olive trees every two years is prohibited, except for irreversible phytosanitary or productive reasons (the ban also applies to plants damaged by war-like operations). This, while on the one hand allowed the safeguarding of Italian olive biodiversity, which is by far the richest, comprising over 630 cultivars—~40% of the world heritage—[49], on the other hand it limited the rejuvenation of olive groves and the modernization of cultivation practices. As an indirect effect, the presence of a vast national germplasm and local varieties that define specific terroirs to which they are historically and culturally strictly tied, have hindered the expansion of the super-intensive model that is based on a limited number of low vigor Spanish (Arbequina and Arbosana) and Greek (Koroneiki) varieties; insofar as the Italian cultivars Urano and Tosca have been proposed as suitable candidates [50,51].

The main consequence of all these occurrences is a high production cost for Italian olive growers, as highlighted in a survey [52] conducted by 9 olive farms in the Emilia Romagna region, concerning the agricultural phase management (water, soil, chemical, and biological treatments, biodiversity, territory and landscape; energy, human, and economic resources), taking into account the cultural and nutritional value of the product. Olive cultivation systems resulted to be defined by obsolete production structures having high costs and low profits, also due to poor mechanization. This economic weakness affects even the social sphere as agricultural workers are generally underpaid and often illegally employed. The authors suggested that the increase in the company surface and the mechanization can contribute to the reduction in production costs.

A quantification of olive oil production costs was realized during an international study by the International Olive Council [53], based on seven different cultivation systems, taking into account orchard density, slope and type of water use (rainfall or irrigation). This survey, involving 15 IOC member countries (including the 5 principal olive producers: Spain, Italy, Tunisia, Greece, and Portugal), took into consideration the costs for olive grove management deriving from fertilization, use of agrochemicals, soil tillage, pruning, harvesting, and water management, as well as indirect and amortization costs for each system to produce one kg of olive oil. Then, the actual distribution of the different cultivation systems per each country was taken into account to arrive at the real weighted cost per country. Globally, the average olive oil production cost of one kilogram of olive oil resulted to be 2.63 €/kg, rising up to 3.95 €/kg in Italy, about ~1.5-fold higher than the cost calculated for Spain, Greece and Portugal and 2-fold higher than that in Tunisia. Accordingly, the average production price of the Italian extra virgin olive oil (EVOO) is generally 1.5/2-fold higher than those recorded in the main competing olive producers. However, the rapid and massive growth of olive oil production, passing from 1,735,000 Mg in the 1995 to 1996 season to 3,207,000 Mg in 2019 and 2020 [54], driven by Spain and with a strong impulse from the North African markets (especially Tunisia), produced an imbalance between supply and demand with consequent price decrease. At the same time, in 2019, important olive oil stocks at EU level pushed the European Commission to approve a private storage aid scheme aimed at stabilizing the market and increasing prices, which, however, has favored almost exclusively Spain where stocks are exceptionally high. Furthermore, according to the EU outlook report for 2019 to 2030 [55], by 2030 the EU’s olive oil production is expected to further grow by around 400,000 Mg (+1.1% per year on average), while wine consumption is projected to decline.

As a result, in Italy production price in October 2020 was 4.05 €/kg of EVOO (averagely 3.76 €/kg during the October 2019 to October 2020 period), this value stood at 2.5, 2.94, and 2.28 €/kg for Spain, Greece, and Tunisia, respectively [56]. These quotes are clearly unsustainable for small farmers, but more in general, for the Italian oliviculture (thus heavily dependent on European financing plans implemented through the Common Agricultural Policy—CAP) that cannot and must not compete with markets prone to overproduction, but it must aim for quality production, promoting socio-economic, and environmental sustainability. In fact, this uneconomical condition, is leading to a slow but steady abandonment of olive groves and prevents small farmers from switching to sustainable management systems, so that olive growing is still often based on rigid schemes of dryland farming and conventional tillage, reflecting in a chemical, physical, and biological soil impoverishment. In fact, repeated mechanical tillage practices to limit the development of weeds adversely affect the indicators of soil stability such as microbial abundance and diversity, organic matter content, porosity, and water stable aggregation [57,58,59] are the cause of significant organic matter (OM) reduction [60]. Conversely, the intensification of olive growing (up to 1800–2000 plants ha^−1^ in super-intensive orchards), together with the massive use of agrochemicals, contribute to soil biological impoverishment and degradation, and water pollution in the “olive agroecosystem” [61,62,63,64,65]. Moreover, an emerging criticality is occurring in super-intensive olive groves, where mechanical harvesting carried out at night has been killing millions of migratory birds sheltering in bushy-shaped olive plants [66]. This is why biological activity and biodiversity are generally higher in semi-abandoned undisturbed orchards or in olive groves managed according to the modern techniques of conservative agriculture [67,68].

## 4. Oliviculture, Common Agricultural Policies (CAP), and Sustainability

After years in which European funding for olive farms was directly linked to production, the 2006–2013 Common Agricultural Policy introduced a the policy of support for income decoupled from production with the establishment of the single payment scheme, linked to sustainable environmental management of the farm (“cross compliance” or “conditionality”). For the olive sector, two specific standards of Good Agricultural and Environmental Conditions (GAEC) have been established regarding (standard 4.3) the “*Maintenance of olive groves and vines in good vegetative conditions*” and (standard 4.5) the “*Prohibition of the grubbing up of olive trees*”. These standards marked the transition to a greener vision of olive growing, but ensured only a minimum level of land maintenance by prevent the spread of weeds and the consequently risk of fires, while the obligations related to the care of the plants were limited to sporadic interventions [69]. Decoupling from production has likely discouraged production in marginal areas (contributing to the decline in production), while the contribution was not linked to any quality certification.

During the 2014 to 2020 PAC, the support policies to the first pillar (“greening”) have been further strengthened, wherever the possibility of considering as greening measures also the so-called “equivalent” components, such as the agro-environmental measures of the rural development programs and environmental certifications, has been foreseen. Olive groves have been exempted from particular greening requirements, so that the specific GAEC standards have been eliminated. Italy has decided, in compliance with Annex IV of Reg. (EU) 1307/2013, not to grant payments if the total amount of direct payments is less than: € 250 for 2015 and 2016; € 300 from 2017. This might have stimulated very small farmers to unite with each other, in order to overcome, through aggregation, the exclusion threshold. However, this phenomenon was probably already underway, insofar as (gross of abandoned olive groves) in the last twenty years, while the number of farms has decreased by almost 290,000 units (from 1,113,000 in 2000 to 902,000 in 2010 to 825,000 in 2020), the olive cultivation surface area has increased by almost 98,000 ha with a consequent increase in the average farm size passing from 0.96 to 1.25 to 1.41 ha [41,47]. An economic incentive has been provided for olive growing with significant economic, social, territorial, and environmental importance: the measure concerns the olive-growing areas adhering to quality systems (Protected Designation of Origin—PDO, Protected Geographical Indication—PGI, organic). In this sense, Italy has the highest number of certified extra virgin olive oils (42 PDOs and 6 PGIs), 4 PDOs for table olives and 75 Traditional Agri-food Products (TAPs, a specific product of a territory tied to the traditional local production) linked to olive products (specifically, 33 extra virgin olive oils, 19 cultivars and typical dishes based on oil or olives). Nevertheless PDOs account on average for only 3.84% of total national production, while organic olive farming accounts for 22% of the whole olive surface [43].

The key elements of the new CAP for the 2021 to 2027 period include a fairer distribution of direct payments, enhancing at the same time environmental and climate ambition, in harmony with the goals set in the European Green Deal. Accordingly, the core component of the European Green Deal is the Farm to Fork Strategy aiming to make food systems fair, healthy, and environmentally-friendly. The Farm to Fork Strategy [70] was designed to encourage and speed the switching to a sustainable food system aimed at having a positive environmental impact (by reducing GHG emissions, adopting sustainable agricultural practices, and promoting the protection of biodiversity), ensuring food availability and safety, promoting fairy trade and a more equitable redistribution of profits.

The development of a framework for sustainable food labeling that covers the nutritional, climate, environmental, and social aspects of products is one the proposals under consideration. Furthermore, since 2009, the European Union carries on sustainable development policies through the EU Sustainable Development Strategy (SDS), and the Environmental Technologies Action Plan, whereas sustainable consumption and production (SCP) is the leading force of the Europe 2020 strategy designed to promote sustainable development.

## 5. Sustainability Certification for the Olive Oil Sector

Environmental and social sustainability are increasingly important elements of attention and decision-making levers for consumers, that are leading to the definition of new purchasing models both at national [71] and international level [72,73,74]. 

In the EVOO sector, several analyses of the factors influencing purchase decisions still indicate (low) price as the principal attribute for consumers’ choice [75,76,77], albeit origin of production, quality (PDO and PGI) and organic certifications play an important role in decision making. This trend toward a greater interest for the origin of production, product certifications, and ethical issues, as well as an increasing willingness to pay premium prices for high-quality products, were further highlighted by other studies in Italy [78,79,80,81,82], Spain [83,84], and Greece [85]. 

The shift to market demands requires a greater commitment by producers that requires a management challenge of the production and organization systems. A standardized evaluation process ensuring compliance with the requirements of the four pillars of sustainability (environmental, socio-economic, cultural, and nutritional) can guarantee a rapid and efficient response in this regard increasing as well as reputation and competitive arguments for the market (e.g. communication, traceability, social responsibility, and technological and cultural investments). In addition, this would entail greater attention to the health properties of EVOO, which can be to all intents and purposes considered a functional food [86].

Apart from the increasing consumers’ sensitivity to environmental, nutritional, and ethical issues, the rationale behind the implementation at national level of a sustainability certification label relies on the economic advantages for the adhering olive companies and their employees that would benefit. First of all, the beneficiaries of the certification would be entitled to access the share of the European funding that Italy allocates to olive-growers adhering to quality systems, regardless of a PDO or PGI recognition. Besides, in 2016 the Italian Government drafted the national olive plan (by article 4 of decree-law no. 51 2015) which provided 32 million euros for the 2015 to 2017 period to support the olive oil sector. A total of 28 million euros of this fund, were intended for a series of interventions linked to the “total” sustainability (protection of traditional varieties, defense of Made in Italy products, aggregation of companies for higher profits, use of sustainable cultivation techniques, etc.). Notwithstanding, the fate of these funds has not been publicly reported and in 2019 the parliamentary question n. 5-01297 (July 9 2019) [87] on the state of implementation of the national olive plan, defined many interventions “still at the starting phase”. The possibility of disbursing funds based on the recognition of a sustainability certification would make the attribution process more streamlined and it would accelerate the payment time, as the funding would be based on the achievement of clear and shared objectives. This would stimulate olive growers to adhere to certification system and to invest in their own farms at significantly reduced costs and would allow a price positioning in the segment of premium quality certified oils. Moreover, compliance with the minimum sustainability requirements would have non-negligible secondary effects such as the reduction in undeclared work, and a more equitable distribution of profits, as well as the protection of a cultural heritage and the defense of the consumers’ health by guaranteeing a superior nutritional level of the olive oil. Lastly, at the international level, the adoption of a sustainability standards could also serve to limit, at least partially, the “Italian sounding” phenomenon, namely the misleading use of images, geographical references, and trademarks evocative of Italy (globally, fake Made in Italy agri-food products have been estimated to worth over 100 billion euros [88]), as well as counterfeiting and adulterations. 

## 6. Sustainability Certifications for the Olive Oil Sector Currently Developed in Italy and the Need for Their Harmonization

In Italy, among the most important sustainability certifications, the National Integrated Production Quality System (SQNPI) is a certification scheme aimed at guaranteeing the technical standards in compliance with the National and Regional Integrated Crop Management Guidelines, and it is recognized on a European level (EU Reg. 1974/2006). The strengths related to the SQNPI are the possibility improve the traceability and the salubrity of the product, to comply with the legal obligations regarding integrated pest management (according to the National Action Plan —NAP— for the sustainable use of plant protection products) and to access public financing measures. The weaknesses identified are the non-simplicity in the path of adhesion and conversion, the SQNPI does not provide for the calculation of any environmental impact indicator and it does not directly consider economic and social aspects; moreover it focuses purely on the agricultural phase of primary production. 

“Made Green in Italy” is the voluntary national scheme for the evaluation and communication of the environmental footprint of products based on LCA provisions. The regulation for the implementation of the Made Green in Italy scheme was approved by Ministerial Decree no. 56 of 21 March 2018, as required by art. 21 of Law no. 221/2015 (“Collegato Ambiente”) containing “Environmental provisions to promote green economy measures and to limit the excessive use of natural resources”. The scheme adopts the PEF methodology, as defined in Commission Recommendation 2013/179/EU, and is aimed at promoting the competitiveness of the Italian production system in the context of the growing demand for products with high environmental qualification on national and international markets. Evaluation concerns only Made in Italy products with environmental performance equal or superior to the reference benchmarks The reference benchmark must always be defined by the proponent of an RCP and calculated as the sum of weighted values. As such, the socio-economic and nutritional components are not considered.

The private certification body Rete Clima offers the emission of sustainability credits and carbon credits due to carbon offset, for Italian olive groves managed according to sustainable cultivation techniques with low environmental impact, to support the reduction in greenhouse gas emissions. The sustainability credits were developed within the Life Olive4Climate Project, and are quantified according to the methodology described in the “Standard for the quantification and certification of sustainability credits deriving from the Sustainable Management of Olive Groves” [89], based on ISO 14067 standard, with validation by the Technical Scientific Committee of the project. The weakness of this certification is that sustainability is considered only in terms of carbon footprint.

The DTP 125 “Sustainable Extra Virgin Olive Oil”, is the first and only sustainability certification for the entire extra virgin olive oil supply chain at a national level. Developed by CSQA certificazioni srl for the Italian olive company Zucchi, this certification arises from the desire to produce EVO oil at the best of known practices to ensure a sustainable product. Furthermore, sustainability is defined according to the model of the three pillars of sustainability, adding a fourth component, the nutritional and health pillar, through which the consumer is assured of a high quality product with more restrictive parameters than those provided for by the applicable laws. This certification, although very complete, lacks the economic and social component. On the other hand, the aforementioned certification system is difficult to interpret and complex to apply and also requires little in-depth analysis of the requirements linked to the landscape and the territory.

Eventually, the Faculty of Agriculture, Food, and Environmental Sciences of the Catholic University of the Sacred Heart of Piacenza, in the scope of the 2014 to 2020 Rural Development Program project of the Emilia-Romagna Region “Development of operational supports for the enhancement and promotion of a highly sustainable olive production chain in Emilia-Romagna–Terre dell’Olivo-”, in collaboration with the Italian Ministry for Environment, Land, and Sea Protection, is currently developing a path for the elaboration of a single standard of sustainability, which can be easily conveyed to all links in the olive-oil supply chain. To achieve the primary objective, the following specific sub-objectives have been set: (i) an assessment of the sustainability of the supply chain through the preliminary analysis of representative companies for olive production and representative mills for processing; (ii) an identification of the areas of intervention and problems to implement good practices; (iii) an analysis and adaptation of existing national sustainability certification schemes.

Therefore, the VIVA certification “the sustainability of viticulture in Italy” was devised in 2011 by the MATTM in collaboration with the Opera Research Center for sustainability in agriculture of the University of the Sacred Heart to improve the sustainability performance of the wine sector through the analysis of four indicators; air; water, vineyard; and territory. In this case, the sustainability diagnosis is faced with two distinct approaches: at the organization level, allowing to carry out an overall assessment of the environmental performance of the company itself, and at the product level, allowing to perform an analysis focused on a specific product. The strengths related to VIVA are the possibility of obtaining product and organization certification; the drafting of improvement plans to be implemented in the two-year period following certification; the issue of an innovative label, which makes sustainability data accessible in a simple, clear, and uniform way; and the training of company technicians on the application of VIVA indicators. The weaknesses of VIVA are identified in the non-consideration of the nutritional and qualitative aspects of the final product, which are very important in the olive-oil supply chain, in the non-traceability of the sustainable product along the supply chain and in its specificity for the wine sector. On the basis of this model, providing for a harmonization of voluntary environmental declarations for the creation of a framework for sustainable labeling for extra virgin olive oil, art. 224-TER of the law decree of 19/05/2020 n-34 should be mentioned. The article extends the sustainability certification of production process to other agri-food supply chains.

## 7. Conclusions

The socio-economic weaknesses of the national oliviculture, are at the root of the loss of competitiveness of the Italian olive companies. It is therefore necessary to move to economically and environmentally sustainable olive grove management systems, linked to a recognized and remunerated context of conservation of territorial heritage and genetic resources and, consequently, of niche productions. The certification of the sustainability of production opens up new market outlets. Italian olive growers must therefore aim for sustainable quality production. That should include in addition to the socio-economic and environmental pillars the nutrition and quality aspects linked to the traditional food culture of the country and for a safe and resilient Mediterranean diet.

According to this analysis, it would be necessary to promote the convergence and harmonization of the certification programs listed. In this way, it could increase the simplicity of the system’s applicability, improve communication with greater sharing of information to consumers, promote adequate planning of training and education courses on sustainability for operators, promote the development of a national territorial network that facilitates technological and cultural changes in the sector. This approach requires public and private financial incentive measures and in parallel, participatory interventions of the production base with companies and entrepreneurs of recognized reputation. From the technical-scientific point of view, the work can then be carried out more effectively since tradition, culture, and scientific knowledge are already available, but unfortunately lost in the absence of a clear operational direction.

## Figures and Tables

**Table 1:** Principal voluntary sustainability standards and labels (VSSL) at the global level in the agri-food sector.

VSSL	Established	Scope of Action
Organic (generic)	1982 (first certification)	Environmental management. Several national and private organic standards exist. At the international level, the International Federation of Organic Agriculture Movements (IFOAM),with over 800 affiliates in 127 countries, provides organic farming standard accreditation and certification service for most non-governmental organic certifying organizations worldwide.
Max Havelaar and Fairtrade	1988	Social and environmental management. Quality *label* to products that have been produced according to principles of fair trade and complying with the guidelines of Fairtrade International.
Rainforest Alliance (RA)	1990	Environmental management. Global certification system for sustainable forest management
UE Ecolabel	1992	Environmental management. UE Ecolabel is the ecological quality trademark of the European Union established in 1992 by Regulation no. 880/92 and is now governed by Regulation (EC) no. 66/2010 in force in the 28 countries of the European Union and in the countries adherents to the European Economic Area—EEA (Norway, Iceland, Liechtenstein).
The Forest Stewardship Council (FSC),	1993	Environmental management. Global certification system for sustainable forest management
Marine Stewardship Council (MSC)	1997	Environmental management. Global standard for sustainable fishing. complying with the 2005 FAO “Guidelines for the Eco-labeling of Fish and Fishery Products from Marine Wild Capture Fisheries”
GlobalG.A.P.	1997	Environmental and social management. GlobalG.A.P. sets voluntary standards for the certification of safe and sustainable agri-food products worldwide and counts more than 400 members among the most important supermarket chains and their major suppliers.

## Data Availability

Data sharing not applicable.
